# Chronic, Recreational Chloroform-Induced Liver Injury

**DOI:** 10.1155/2018/1619546

**Published:** 2018-09-10

**Authors:** Emily A. Minor, Mackenzie S. Newman, Justin T. Kupec

**Affiliations:** ^1^Department of Physiology and Pharmacology, West Virginia University School of Medicine, Morgantown, WV, USA; ^2^Department of Digestive Diseases, West Virginia University School of Medicine, Morgantown, WV, USA

## Abstract

Historically used as an anesthetic, chloroform is a halogenated hydrocarbon that is associated with central nervous system depression, arrhythmias, and hepatotoxicity. It is no longer used clinically, but accidental and intentional poisonings still occur. We report a case of chronic chloroform abuse leading to severe hepatotoxicity in a 26-year-old male graduate student. The patient presented to the emergency department with a three-day history of abdominal pain, dehydration, and scleral icterus. He drank several beers the night before the onset of symptoms, but denied taking acetaminophen, ibuprofen, or other drugs. An extensive work-up revealed an aspartate aminotransferase (AST) of 13,527 U/L and alanine aminotransferase (ALT) of 8,745 U/L, but the cause of his liver injury could not be determined. It was not until many months later that the patient admitted to inhaling chloroform in the weeks leading up to his illness.

## 1. Introduction

Chloroform is no longer used to induce anesthesia, but it is commonly used as a solvent in laboratory settings and poisonings still occur. There are several cases that describe chloroform-induced hepatotoxicity following suicide attempts, but this is the first known report of chronic, recreational chloroform abuse [1, 2]. The patient was a graduate student who inhaled chloroform obtained from his laboratory for several months leading up to his hospitalization. He did not disclose that initially because he did not feel comfortable discussing his drug use. Herein we describe the clinical presentation of chronic exposure to chloroform and highlight the importance of establishing a strong rapport with patients in order for there to be an open and honest dialogue about substance abuse.

## 2. Case Presentation

A 26-year-old male graduate student presented to the emergency department with a three-day history of nausea, vomiting, and abdominal pain. He additionally complained of dark urine with sediments, dehydration, and scleral icterus. Laboratory evaluation revealed an aspartate aminotransferase (AST) of 13,527 U/L, alanine aminotransferase (ALT) of 8,745 U/L, and ferritin of >40,000 ng/mL. His total bilirubin was 5.5 mg/dL and international normalized ratio (INR) was 1.95, suggesting a chronic liver injury (Table 1). The patient reported drinking four to five beers the night before the onset of symptoms but denied taking acetaminophen, ibuprofen, or other drugs. He was given intravenous saline and admitted to the hospital for further evaluation.

Given his presentation and significant transaminase elevation, drug-induced hepatitis or viral hepatitis was suspected, while ischemic hepatopathy was unlikely. Right upper quadrant ultrasound was unremarkable. Acetaminophen, salicylate, and tricyclic levels were negative. Mononucleosis, human immunodeficiency virus, cytomegalovirus, antinuclear antibody, and viral hepatitis panel were also negative. Herpes simplex and varicella zoster IgG were positive, likely due to prior infection or immunization.

He remained stable during hospitalization and his liver enzymes began to trend downward, so he was discharged two days after admission. At six-week gastroenterology follow-up visit, his liver enzymes had returned to normal and he reported complete resolution of symptoms. The remainder of the pending laboratory evaluation (smooth muscle antibodies and hemochromatosis testing) returned negative.

The etiology of this patient's hepatitis remained unknown until many months later he admitted to inhaling chloroform. He described recreational huffing, to the point of unconsciousness, twice weekly over several months. This information was not volunteered at the time of presentation because the patient was embarrassed and did not feel comfortable discussing substance abuse with his physicians.

## 3. Discussion

Chloroform is a volatile organic compound that is commonly used as a solvent, cleanser for plastic compounds, and acrylic adhesive [3]. Exposures can occur accidentally or intentionally in various occupational settings including cleanrooms, laboratories, and factories. Several cases describe hepatotoxicity from acute chloroform exposure, but we provide the first known report of severe hepatitis resulting from chronic, recreational chloroform abuse. The liver enzyme elevations seen in our patient were significantly higher than those following acute consumption (AST 1,160 U/L, ALT 3,560 U/L) and suicide attempts (AST 1,513 U/L, ALT 2,717 U/L) [1, 2]. Regardless of the intent of use, liver injury is largely the result of phosgene, a toxic metabolite formed via cytochrome P450 2E1 [4]. Phosgene participates in acylation reactions with proteins, carbohydrates, and lipids, which depletes glutathione stores and leads to severe oxidative damage and ultimately hepatocellular steatosis and necrosis [5]. Concomitant alcohol consumption, as seen in our patient, magnifies this effect via cytochrome P450 enzyme induction [6].

The patient experienced significant transaminase elevation, but physicians could not determine the cause because they were missing key information. People with access to chemicals, either professionally or educationally, should receive a thorough occupational history and any exposures should be classified and quantified. For example, chloroform poisoning should be considered in graduate students, cleaners, and factory workers with unexplained liver injury. If questions arise about treatment, physicians should then reference the material safety data sheets (MSDS). In addition, completion of a social history to establish comorbid conditions or behaviors will allow accurate guidance for further testing or care.

The nonspecific nature of the patient's symptoms and his undisclosed drug use made it hard to establish the correct diagnosis. People who use illicit substances are often reluctant to divulge such information because of the stigma associated with drug use. In order to overcome this, healthcare providers must establish a strong, professional rapport with patients so that there can be an open and honest dialogue. Additionally, a thorough occupational history can help uncover potential exposures. In conclusion, chloroform and other halogenated hydrocarbons can lead to severe hepatotoxicity and should be considered in certain populations who present with unexplained liver injury.

## Figures and Tables

**Table 1:** Laboratory findings.

	Reference range	Day of admission (14:00)	Day of admission (20:00)	Hospital day 1	Hospital day 2	6 week follow-up
AST	8-48 U/L	13,527	9,319	5,137	2,140	23
ALT	0-55 U/L	8,745	5,985		3,994	29
Bilirubin (Total)	0.3-1.3 mg/dL	5.5	3.9	7.4	7.6	0.7
Bilirubin (Conjugated)	0.0-0.3 mg/dL	3.5	2.6	4.7	5.4	0.3
LDH	125-220 U/L	3250				
Prothrombin Time	8.7-13.2 sec	21.7		22.2	16	
INR	0.8-1.2	1.95		1.99	1.45	
Ferritin	20-300 ng/mL			> 40,000		503
Ammonia	15-50 umol/L	120			47	
Urine Protein	0 mg/dL	30				
Urobilinogen	0 mg/dL	4				
